# New paths for modelling freshwater nature futures

**DOI:** 10.1007/s11625-023-01341-0

**Published:** 2023-07-03

**Authors:** Lilith Kramer, Sven Teurlincx, Brenda Rashleigh, Annette B. G. Janssen, Jan H. Janse, Kate A. Brauman, Csaba Földesi, Dianneke van Wijk, Lisette N. de Senerpont Domis, Sopan D. Patil, Parinaz Rashidi, Perrine Hamel, James Rising, Wolf M. Mooij, Jan J. Kuiper

**Affiliations:** 1 Department of Aquatic Ecology, Netherlands Institute of Ecology (NIOO-KNAW), Wageningen, The Netherlands; 2 Aquatic Ecology and Water Quality Management Group, Wageningen University and Research, Wageningen, The Netherlands; 3 Department of Freshwater Ecology and Water Quality, Deltares, Delft, The Netherlands; 4 U.S. Environmental Protection Agency, Office of Research and Development, Narragansett, RI USA; 5 Water Systems and Global Change Group, Wageningen University and Research, Wageningen, The Netherlands; 6 PBL Netherlands Environmental Assessment Agency, The Hague, The Netherlands; 7 Global Water Security Center, University of Alabama, Tuscaloosa, AL USA; 8 Pervasive Systems, Faculty of Electrical Engineering, Mathematics and Computer Science, University of Twente, Enschede, The Netherlands; 9 Department of Water Resources, Faculty of Geo-Information Science and Earth Observation, University of Twente, Enschede, The Netherlands; 10 School of Natural Sciences, Bangor University, Bangor, UK; 11 Asian School of the Environment and Earth Observatory of Singapore, Nanyang Technological University, Singapore, 639798 Singapore; 12 School of Marine Science and Policy, University of Delaware, Newark, USA; 13 Stockholm Resilience Centre, Stockholm University, Stockholm, Sweden

**Keywords:** Freshwater ecosystems, Scenarios, Modelling, Nature Futures Framework, Value perspectives, IPBES

## Abstract

Freshwater ecosystems are exceptionally rich in biodiversity and provide essential benefits to people. Yet they are disproportionately threatened compared to terrestrial and marine systems and remain underrepresented in the scenarios and models used for global environmental assessments. The Nature Futures Framework (NFF) has recently been proposed to advance the contribution of scenarios and models for environmental assessments. This framework places the diverse relationships between people and nature at its core, identifying three value perspectives as points of departure: Nature for Nature, Nature for Society, and Nature as Culture. We explore how the NFF may be implemented for improved assessment of freshwater ecosystems. First, we outline how the NFF and its main value perspectives can be translated to freshwater systems and explore what desirable freshwater futures would look like from each of the above perspectives. Second, we review scenario strategies and current models to examine how freshwater modelling can be linked to the NFF in terms of its aims and outcomes. In doing so, we also identify which aspects of the NFF framework are not yet captured in current freshwater models and suggest possible ways to bridge them. Our analysis provides future directions for a more holistic freshwater model and scenario development and demonstrates how society can benefit from freshwater modelling efforts that are integrated with the value-perspectives of the NFF.

**Graphical abstract:**

**Figure:**
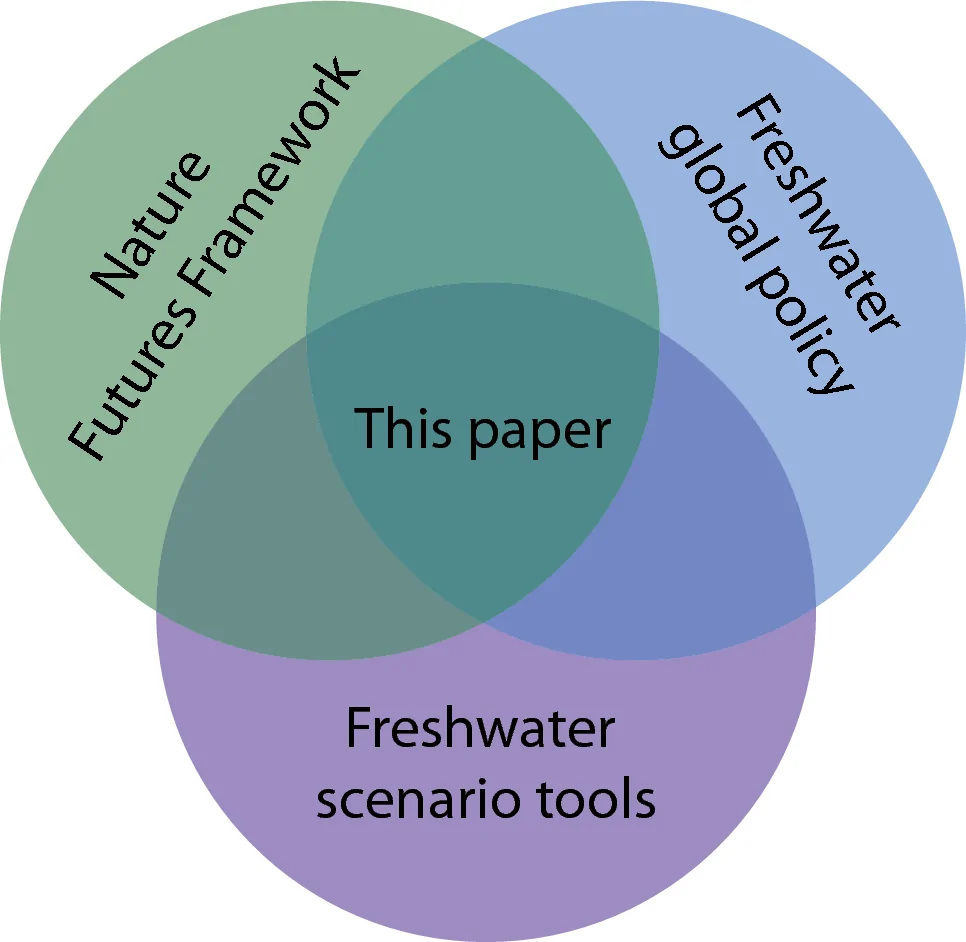

## Introduction

The freshwater biome—comprising rivers, lakes, ponds, freshwater wetlands, as well as human-made systems such as drainage ditches and quarry lakes—is exceptionally rich in biodiversity (Román-Palacios et al. 2022). Although freshwater systems (including wetlands) cover ~ 8% of the world’s land surface area, they harbour about a third of all vertebrates and nearly half of all fish species (Lehner and Döll 2004; Balian et al. 2008; Reid et al. 2019). This biodiversity underpins critical freshwater ecosystem services (Geist 2011; Janssen et al. 2021). Ultimately, the well-being of humanity depends upon sustaining freshwater ecosystems (Albert et al. 2021). It is therefore alarming that freshwaters are perhaps the most imperilled ecosystems on the planet (Reid et al. 2019; WWF 2020), suffering from multiple interacting and accumulating stresses including climate change, water over-extraction, overexploitation, pollution, invasive species and infrastructure development (Reid et al. 2019). It is estimated that almost a third of the monitored vertebrate freshwater species are threatened with extinction (Collen et al. 2014; WWF 2020), and their populations globally have declined by 81% since 1970 (McRae et al. 2017). In addition, freshwater ecosystems are under-represented among the world’s protected areas and/or lack protected areas in their upstream catchment (Abell et al. 2017).

At the same time, freshwater ecosystems and their biodiversity remain understudied and underrepresented in global environmental assessments that inform global environmental governance (UN Environment 2019; IPBES 2019a). Indeed, freshwater ecosystems are often not addressed explicitly, but lumped together with terrestrial ecosystems (van Rees et al. 2021). Freshwater biodiversity is particularly poorly represented in the scenarios and modelling tools that support these global assessments (IPBES 2016). However, evidence shows that explicitly integrating the needs of freshwater species into conservation strategies increases the overall benefits to freshwater species vastly, with almost no loss to the terrestrial species benefits (Leal et al. 2020). There is a risk that the neglect of freshwater ecosystems will be reflected in gaps in the development and assessment of transformative pathways to sustainable futures, and directly would undermine the viability of these pathways.

In contrast to the poor representation of freshwater ecosystems in global scenarios and models, we recognize that a wealth of tools, and associated communities of practice, exists for environmental assessments of freshwater ecosystems at the local, regional and catchment scales (e.g. to comply with the EU Water Framework Directive 2000). Since the 1920s, and especially since the introduction of the personal computer in the 1970s, many disciplines have developed models to understand freshwater ecosystems, perform scenario analysis and inform policy (Jørgensen 1995). The models available today specialize in various freshwater ecosystems, focus on different ecosystem elements, and use a wide array of modelling approaches (see Janssen et al. 2015 for an overview). Examples of models applied in freshwater management are SWAT (Krysanova and White 2015), PCLake (Janse 2005; Yang et al. 2022) and the Water Framework Directive Explorer (Visser et al. 2022).

Recently, the taskforce on scenarios and models of Intergovernmental Science-Policy Platform on Biodiversity and Ecosystem Services (IPBES) proposed the Nature Futures Framework (NFF) to advance the role of scenarios and models in guiding global policy (Pereira et al. 2020), based on the knowledge gaps and needs identified by the IPBES thematic assessment report on scenarios and models (IPBES 2016; Kok et al. 2017; Rosa et al. 2017). The NFF provides a more pluralistic approach than the widely adopted conservation and ecosystem services approach; it provides guidance for the development of diverse social-ecological scenarios by the broader research community and aligns well with the IPBES approach to Nature’s Contributions to People (IPBES 2019a). The framework engages people’s diverse and plural values of nature, using three broad value perspectives of nature*—Nature for Nature*; *Nature for Society*; and *Nature as Culture*—as entry points. The framework further distinguishes itself by focusing on desirable futures for people and nature, and the transformative changes needed to achieve them. As such, especially when quantified with models, nature futures scenarios may be used to substantiate and develop pathways toward the internationally agreed 2050 Vision “Living in harmony with nature” of the Convention on Biological Diversity (CBD), operationalize the ‘Sustainable Freshwater Transition’ as outlined by the Global Biodiversity Outlook 5 (Secretariat of the Convention on Biological Diversity 2020), and support the ‘Emergency Recovery Plan’ to bend the curve of freshwater biodiversity loss (Tickner et al. 2020).

Maasri et al. (2022) listed the development of nature futures scenarios for IPBES as one of the key priorities for advancing freshwater biodiversity research. However, while there is an increasing number of NFF studies presented in the literature (e.g. see this issue), to date there are limited applications focusing on freshwater (but see e.g. Resende et al. 2020, who present a conceptual model to assess the impact of anthropogenic drivers on water-related ecosystem services in the Brazilian Cerrado). In this paper, we respond to the need for more actionable knowledge of how the NFF can be used to develop and quantify scenarios of freshwater futures, looking into the indicators and models that could be harnessed. Specifically, we explore the following interconnected questions: (1) how freshwater ecosystem concepts align with the NFF; (2) what the NFF means for developing scenarios for freshwater systems; and (3) what models are needed to quantify these scenarios. By connecting freshwater modelling efforts and communities to the NFF, we foresee the potential for increasing freshwater representation in global policy and integration of quantitative environmental assessment tools within the NFF. We end with a call to connect and mobilise communities of practice that are not yet connected to IPBES assessments.

## NFF for freshwater ecosystems

### Unpacking the value perspectives on freshwater nature futures

**Fig. 1:**
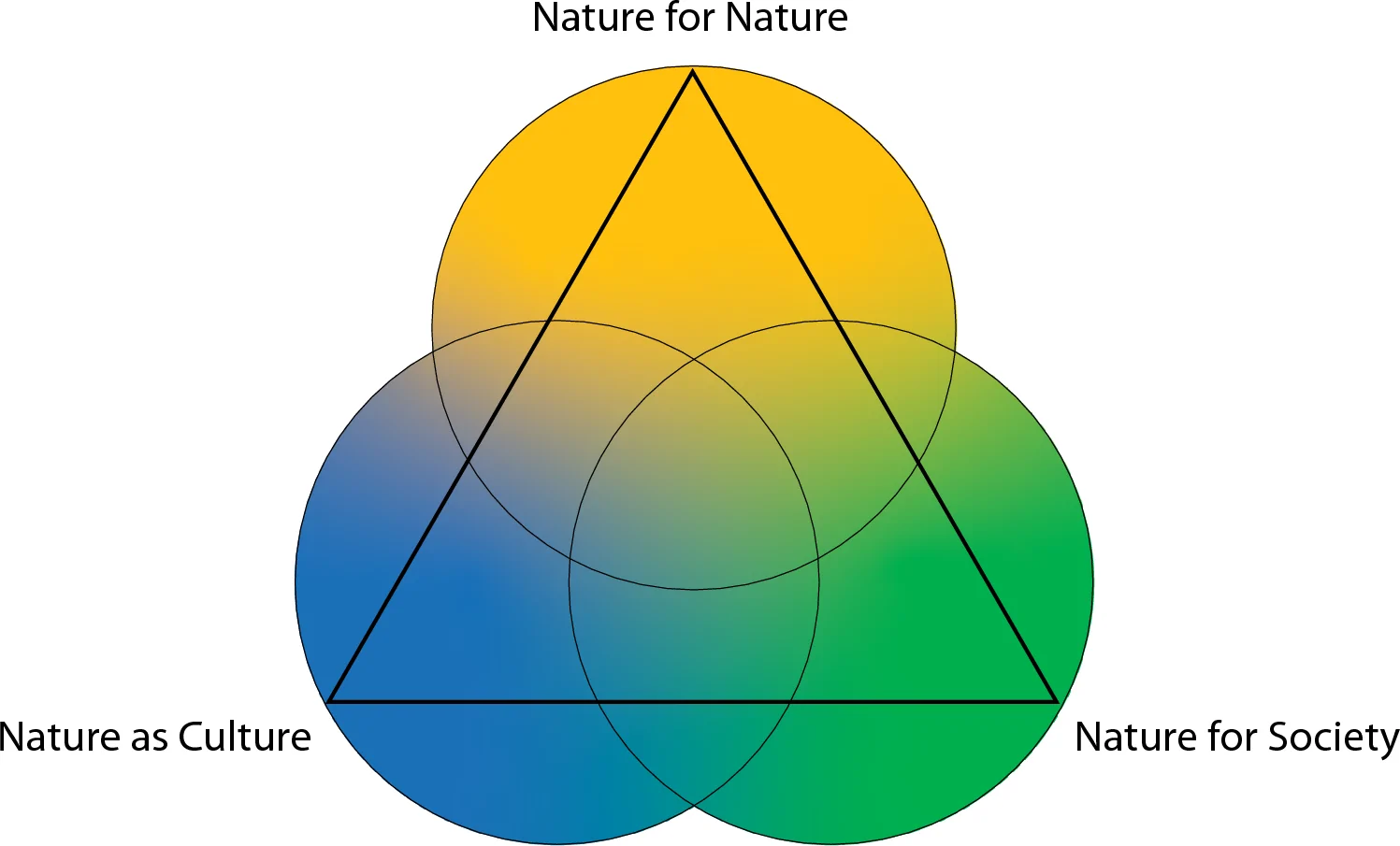
Visualisation of the Nature Futures Framework (Source: PBL 2018)

The NFF was proposed as a flexible tool to catalyse the development of diverse social-ecological scenarios that describe positive futures for nature and people (Pereira et al. 2020). It positions the three broad value perspectives (Table 1) in the angles of a triangular figure (Fig. 1), thereby opening up an interior space for exploring the diversity and plurality of people’s desired relationship with nature as a basis for scenario development and modelling (Ibid). The three value perspectives provide reference points that are applicable across spatiotemporal scales and regions, offering a simple structure for consistency in the scenarios and models that use it. At the same time, the NFF allows for exploring the plurality of desirable people-nature relationships in resonance with local realities, based on which an infinite number of scenarios can be co-created. Such collaborative knowledge production is key to enabling transformative change toward sustainability (Schneider and Rist 2014; Hakkarainen et al. 2022). An important first step in operationalizing the NFF for freshwater ecosystems is to obtain a better understanding of how the value perspectives apply to freshwater ecosystems.

**Table 1:** Generic summary overview of the NFF value perspectives (c.f. Pereira et al. 2020; IPBES/TF/SCN/2021/1/2)

NFF value perspective	Nature for Nature	Nature for Society	Nature as Culture
Summary description	In which nature has value in and of itself. Nature maintains its ability to function autonomously, and the preservation of nature’s diversity and functions is of primary importance	In which nature is primarily valued, and sustainably managed for the benefit of humans	In which humans are perceived as an integral part of nature, where societies, cultures, traditions and faiths are intricately intertwined with nature, and relational values, such as those reflecting cultural identities and ways of life, are dominant
Prevailing value type *IPBES/4/INF/13*	Intrinsic values	Instrumental values	Relational values

Here, we take a literature-grounded approach to unpack the value perspectives, by compiling a non-exhaustive overview of existing concepts and approaches from freshwater ecosystem science and conservation. We think that these existing concepts and approaches can be leveraged to facilitate collaborative connections with relevant research communities and reduce the risk of “reinventing the wheel” (Mooij et al. 2010). Visioning is another powerful way of demonstrating how people’s values and preferences can give shape to fundamentally different future waterscapes (cf. Mansur et al. 2022 who created narratives and illustrations of visions of urban nature futures). Although we take a complementary approach, we have included the freshwater future visions from Boeren et al. (2021) as we think such images can be inspirational (Box 1).

The order in which we present each of the value perspectives in this paper will be *Nature for Nature*, *Nature for Society* and *Nature as Culture*. This order is similar to the one used in earlier representations of the NFF (Pereira et al. 2020). Although an order can represent a preference, e.g. with the item mentioned in the first place being the best, we would like to stress that each value perspective we mention is equally valid.

Box 1. Illustrations of future visions representing Nature for Nature (left), Nature for Society (centre), and Nature as Culture (right) (from Boeren et al. 2021, with permission from Roel Riphagen) A group of MSc students was commissioned to envision positive freshwater futures for the Netherlands in 2050 using the NFF in a consultancy course at Wageningen University and Research. The project involved building a conceptual framework on humans and their relations with freshwater ecosystems and the application of the NFF. Based on this, three distinct visions were created—one for each extreme interpretation of a value perspective. Subsequently, policy options were identified that can steer current trajectories towards the visions. The visions are:

Flowing freely—A Nature for Nature vision, in which humans live in densely populated cities, and through large restoration projects they have freed up space for free-roaming and interconnected freshwater systems. Cities provide a vital link in the freshwater system via green–blue solutions and humans only interfere to maintain the ecological flow of freshwater systems.

Directing the flow—A Nature for Society vision, in which humans live in a circular society that optimises the delivery of ecosystem services while ensuring ecosystem functioning. Through multifunctional ecosystem service delivery and technological improvement, humans reduce their pressures on freshwater systems and integrate freshwater into people’s everyday lives.

Becoming the flow—A Nature as Culture vision, in which freshwater is part of daily life. It flows through the cities, offers space for housing and farming, and represents the cultural foundation of many communities. Through education and awareness programmes, people reconnect with water and develop a caring relationship with it, recognising it as the source of life. In the illustration, anthropomorphic water figures are becoming the flow; thereby representing the strong connection between people’s identities and the way freshwater flows through the landscape.

**Figure:**
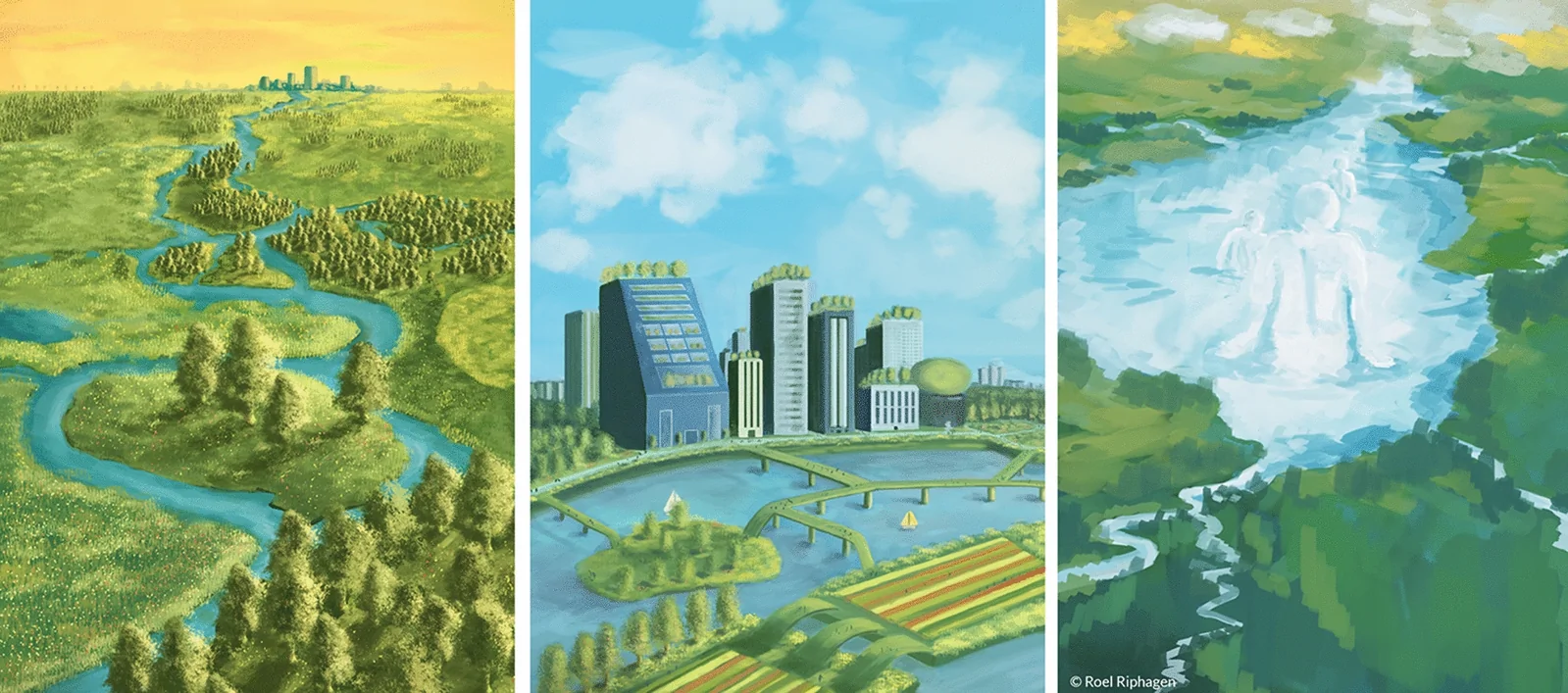

#### Nature for Nature concepts

The Nature for Nature perspective embodies intrinsic values of nature, i.e., valuing nature in and for itself. As such, the protection of freshwater bodies in *freshwater-protected areas* (Saunders et al. 2002; Gardner and Davidson 2011) is one of the key concepts of the Nature for Nature value perspective. However, given the key role of connectivity in defining freshwater ecosystems (Saunders et al. 2002; Teurlincx et al. 2019), the effectiveness of area-based approaches is limited. The *natural flow regime,* a concept introduced by Poff et al. (1997), describes how the ecological integrity of rivers and streams is dependent on dynamic and variable water regimes. A broader concept, looking towards the restoration of ecosystems through active or passive removal of human influence, is *rewilding*. For freshwater ecosystems, this could mean restoration of the natural flow regime over a great distance downstream through the removal of dams, dikes, or other man-made structures (Mitsch 2013; Rideout et al. 2021), or the reintroduction of ecosystem engineers, such as beavers (Law et al. 2017). Recently, Turak et al. (2017) explained how *Essential Biodiversity Variables* are useful for measuring change in global freshwater biodiversity, in terms of genetic composition, species populations, species traits, community composition, ecosystem structure, and ecosystem function*.* A legal concept to protect freshwater ecosystems is *environmental personhood*. This concept designates environmental entities, such as rivers and lakes, the status of a legal person, thus giving legal rights, protections, privileges, responsibilities, and legal liability (Cano Pecharroman 2018). The issue of the fact that nature cannot speak for itself is handled through the selection of human representatives. A more radical point of view on the legal system is found in the work of Celermajer et al. (2021) on the idea of *multispecies justice*, a concept that rejects the idea that humans are separate and unique in comparison to all other species and that could redefine how we approach justice today.

#### Nature for Society concepts

The Nature for Society perspective promotes instrumental values of nature, i.e., valuing nature for its utilitarian benefits to people. A key concept underpinning this perspective is *freshwater ecosystem services,* including provisioning services, such as the supply of freshwater fish for food, regulating services, such as recharge of drinking water supplies and regulation of water quality, and non-material services including water-based recreation and tourism (see Janssen et al. 2021; Vári et al. 2022 for recent overviews). A closely related but more inclusive concept is *Nature’s Contributions to People* (NCPs), of which the generalised perspective on regulating and material NCPs is particularly relevant here (Díaz et al. 2018). The *environmental flows* concept describes “the quantity, timing, and quality of freshwater flows and levels necessary to sustain aquatic ecosystems which, in turn, support human cultures, economies, sustainable livelihoods, and well-being” (Arthington et al. 2018). Linked to sustainable use is the *Maximum Sustainable Yield* (MSY), defined as the maximum catch that can be removed from a (fish) population over an indefinite period (Maunder 2008). *Ecological resilience*, and derived concepts like *safe-operating space* (Carpenter et al. 2017) and *critical loads* (Janse et al. 2008), are used to determine exploitation levels beyond which undesirable changes occur. Freshwater flows and associated contributions to people can be strongly affected by the watershed landscape. The NCPs provided by the landscape to freshwater systems, encompassing the ways that nature regulates the quantity, quality, location, and timing of flows, are addressed in *hydrologic ecosystem services* literature (Brauman et al. 2007).

In the urban context, the concepts of *Blue Infrastructure* (Andersson et al. 2019) and *Blue spaces* (Garrett et al. 2019) are gaining traction, referring to freshwater systems in the landscape, and their functional connections, that have the potential to provide ecosystem services like moderating urban heat waves. The closely related topics of *Nature-based solutions, nature-based water management,* and *green infrastructure* encompass projects that harness landscape processes to improve the management of water for various benefits, such as flood risk reduction (World Water Assessment Programme/UN-Water 2018).

#### Nature as Culture concepts

The Nature as Culture perspective considers humans as an integral part of nature and values the reciprocal relationship between people and nature. It recognizes that multiple worldviews exist and that they are underpinned by values that are neither intrinsic nor instrumental, as is clear from engaging Indigenous Peoples and Local Communities (Pereira et al. 2020; IPBES 2022). Water is central to many religious, spiritual, and traditional practices and other cultural aspects that shape people’s identities. For indigenous peoples, water bodies are often sacred and ancestral (Jackson and Barber 2015; Latchmore et al. 2018; Machado 2020). However, western conceptualizations of freshwater ecosystems generally struggle to address local and indigenous views, values, and knowledge systems. Indeed, “How can we broaden the current models and orthodoxies at the science-policy interface to integrate worldviews from indigenous and multicultural understandings?” is one of 25 essential research questions to inform the protection and restoration of freshwater biodiversity posed by Harper et al. (2021). Useful concepts that emphasise the combined cultural and natural elements of water do exist. *Cultural Keystone Species* are species that critically shape the cultural identity of indigenous peoples, as reflected in the fundamental roles these species have in diet, materials, medicine, and/or spiritual practices (Garibaldi and Turner 2004; Noble et al. 2018). Related to environmental flows, the *cultural flows* concept focuses on managing flows in ways that recognize, respect, and support cultural ways of life (Lokgariwar et al. 2014; Magdaleno 2018). Also relevant are the *‘context-specific perspectives’* on NCPs (Díaz et al. 2018) and the *non-material contributions of nature*, such as spirituality, but also cultural dimensions of boating, angling, and ice-skating. These concepts are in principle not restricted to indigenous communities but may also apply in ‘western’ or urbanised societies. More generally, *sense of place* (Murphy et al. 2019) and *biocultural approaches* (Johnston 2013) to understanding people's relationship with freshwater systems align with the Nature as Culture perspective.

#### Overlapping concepts

Many of the presented concepts are not bound to a single value perspective. The environmental flows concept is used to promote the integrity of ecosystems, and to support human cultures, economies, sustainable livelihoods, and well-being; thereby addressing all three value perspectives (Arthington et al. 2018). Moreover, through the concepts, the value perspectives are able to reinforce each other. For example, giving rights to nature can be a vehicle for recognising indigenous peoples' relationships with natural entities (Cano Pecharroman 2018). Establishing freshwater-protected areas can increase both protein and cash returns to fisheries (Hannah et al. 2019). Riverine reserves created by local/indigenous communities have markedly increased the richness, density, and biomass of fish relative to adjacent areas (Koning et al. 2020). Bremer et al. (2018) revealed the overwhelming importance of relational values underlying ‘upstream’ participation in Payments for Watershed Services projects. Therefore, each concept could also have possible co-benefits and synergies and may be used in the creation of scenarios where multiple values are enhanced.

### Quantifying freshwater nature futures

After unpacking the three value perspectives to better understand how the NFF opens a space for representing a diversity of desirable freshwater nature futures, this section looks into how to conduct quantitative assessments of those futures, not least to identify possible actions to get there. Such assessments of the future typically involve the development of scenario storylines and quantifying these scenarios with models. We outline an approach for designing scenarios and selecting models for freshwater nature futures, by reflecting on the key components and giving examples of freshwater models that are already available to us.

Although we mention scenarios before models, it is important to keep in mind that the creation of scenarios and running of models typically involve an iterative process. Conducting a quantitative modelling exercise requires one to become specific about inputs, outputs, and processes, possibly pointing to gaps in scenario storylines. Furthermore, the logic of dynamically interacting mathematical equations challenges scenario assumptions, while model outcomes enrich storylines. Nevertheless, it is advisable to start with scenarios, so as to not be limited by what (existing) models can do. Indeed, it might be that essential elements of the scenarios are not yet in any model and have to be researched and developed.

#### Scenarios

Scenarios are descriptions of how the future may unfold. IPBES (2016) uses a classification of scenario types that can be linked to different phases of the policy cycle.

*Exploratory scenarios* examine a range of plausible futures based on potential changes in direct and indirect drivers of change, making them particularly relevant when faced with high levels of uncertainty. As such, exploratory scenarios are often used to assess the consequences of environmental change, to raise awareness of future challenges and support agenda-setting. Typical examples are the Representative Concentration Pathways (RCPs) and the Shared Socio-Economic Pathways (SSPs) developed by the climate community, which have been used to, for example, explore possible futures for the freshwater security of the country of Jordan (Yoon et al. 2021), global nutrient emissions to waters (Beusen et al. 2022), and the climate variability of fish (Dahlke et al. 2020; Barbarossa et al. 2021).

In *policy-screening scenarios*, the effects of alternative policy or management options are forecasted and compared with a predefined policy-relevant variable. For example, alternative scenarios of agricultural best management practices for the Lake Erie watershed were used to evaluate their effect on nutrient loading into Lake Erie (Bosch et al. 2013).

*Target-seeking scenarios* start from an agreed-upon future target, after which possible pathways towards that desired outcome are explored. This means thinking in measures that can be taken in the present day to obtain the target(s), recognizing that adaptive management can be used to enhance outcomes (Kingsford et al. 2011). It is also possible to approach target-seeking scenarios in reverse, working from the future towards the present. This technique is also known as ‘backcasting’ (Dreborg 1996; Paehlke 2012). An example of a target-seeking scenario is the adaptive plan for long-term water management of the Rhine Delta in the Netherlands developed using the “Dynamic adaptive policy pathways” approach (Haasnoot et al. 2013).

As one of the strengths of the NFF lies in its definition of a desired future (i.e. target), at first glance target-seeking scenarios seem the only way to approach the visions of the future of the NFF. However, although policy screening scenarios do not work directly towards the target, they are still useful to see which policies bring the present the closest to the desired future. Even exploratory scenarios remain useful, for example, to stress-test target-seeking scenarios (see van Vliet and Kok 2015).

Durán et al. (2023) show how different scenario storylines can be developed based on the NFF. The NFF can also be combined with existing frameworks and methods for scenario creation. For example, in a participatory workshop in a new National Park the NFF was applied together with the Three Horizons Framework and the Sustainable Development Goals to collaboratively think about what desirable futures could look like, and assess their potential contribution to sustainable development (Kuiper et al. 2022).

While it is helpful to stick to generic and broad ideas at the start of scenario development, these ideas need to become quantifiable at the moment the scenarios are applied to models. For example, a biodiverse future sounds attractive but this idea needs refining to be able to address relevant (model) questions such as what species groups we are referring to, and in what quantity we consider them to be sufficient in our desired future. As it is sometimes difficult to understand what can be modelled and what cannot, involving modelling experts during scenario development assists in bringing the scenarios closer to model feasibility (Volkery et al. 2008). Additionally, finding the right elements to quantify might prove challenging, especially when taking into account that not every NFF value perspective is commonly included or modelled yet (Kim et al. 2021), let alone the value perspectives represented by the plural interior space of the framework. Therefore, we provide starting points for quantifiable elements (indicators) for each of the three value perspectives in Box 2.

Box 2. Indicators of freshwater nature futures *Nature for Nature indicators*

There are many elements that could be incorporated into a scenario to monitor the impact of actions on the intrinsic value of freshwater ecosystems. One that is widely used is the area size, in the form of the proportion of area (%) protected (Nel et al. 2007; Dorji et al. 2020) or the area of intact ecosystems within a country. Besides that, one could monitor if specific species are positively influenced by a certain measure. For example, by checking if the number of individuals is increasing (Reid and Morris 2017) or by checking if the ecosystem that supports a certain species is still functioning (Fisk II et al. 2014). Additionally, one could use a biodiversity index, such as the biological integrity index, or the mean species abundance, to monitor if foreseen actions change the overall biodiversity in an area (Janse et al. 2015). A set of Essential Biodiversity Variables (EBVs) have been proposed to harmonise different approaches to measuring and monitoring the state of nature (Turak et al. 2017; Jetz et al. 2019).

*Nature for Society indicators*

The wide range of regulating and material benefits of freshwater ecosystems to people can be evaluated using both biophysical indicators and human impact indicators (Brauman et al. 2020). For example, freshwater systems provide a critical material benefit for food production. This benefit could be quantified using biophysical indicators such as total fish biomass, fish landings, or fish biodiversity (Lynch et al. 2016). Food production from freshwater systems could also be quantified using human impact indicators such as fish protein available per capita, nutritional status of fisherfolk, and income from fishing (Lynch et al. 2016). Monetary value is just one of many indicators of the benefit of nature for society. Quantifying benefits in economic terms can be useful, especially when it allows for the integration of ecological studies with other monetary-based policy assessments. However, although there is a wide range of approaches to monetization, economic approaches in practice frequently fail to capture the full suite of benefits and drawbacks of nature to society (IPBES 2022). Regulating benefits are most often quantified using biophysical indicators because human well-being is closely associated with environmental conditions, and environmental conditions are frequently dominated by drivers other than ecosystem services. For example, the risk of floods can be reduced through the preservation of upstream floodplain wetlands (Acreman and Holden 2013). Human well-being might be measured as health impacts from pollutants in water sources or lost revenue from expanding coastal dead zones (IPBES 2019b). The importance of indicators across supply, demand, and value has been highlighted by GEO BON’s work on Essential Ecosystem Services Variables (Balvanera et al. 2022). For global studies, the number of people in a watershed may be used as a large-scale proxy indicator (e.g. Chaplin-Kramer et al. 2019).

*Nature as Culture indicators*

Freshwater systems also provide materials and environments central to relational values and non-material benefits such as learning, experience, culture, and identity (Lynch et al. 2016). Because these values and benefits tend to be highly dependent on cultural context, indicators are most often locally specific and a large body of literature is developing to support plural valuation approaches (Dasgupta 2021; IPBES 2022). A way to measure cultural values is through locally developed indices, such as The Cultural Health Index (CHI) for freshwater (Harmsworth et al. 2011; Moggridge et al. 2022). The CHI can be generalised into three pillars: the cultural importance of the site, the importance of the site for cultural or spiritual resource use, and the cultural requirements of the local or regional water system. Each of these three pillars can in turn be used to define a set of more concrete quantitative indicators. For example, the *cultural importance of the site* can become the presence of sacred rivers (Lokgariwar et al. 2014); the *importance of the site for cultural or spiritual resource use* can become fishing of cultural keystone species (De Alessi 2012), or the number of people that can ice skate (Knoll et al. 2019); and *cultural requirements* can become maintaining historical conditions of flow.

A more specific example of well-defined Nature as Culture indicators can be found in Mãori natural resources management in New Zealand (Harmsworth et al. 2016) where multiple indexes have been developed aimed at including and weighing the cultural importance of Mãori heritage in decision-making around fisheries, nature, and water management. In Bali, studies of the traditional *subak* system—whereby local “water temples” manage irrigation and affect the ecology of entire landscapes—also illustrate possible indicators of relational values associated with Nature as Culture (Lansing 1987).

#### Models

Models are critical tools to generalise, interpret and extrapolate links between drivers of change and an indicator of interest (IPBES 2016). Important elements for modelling systems are the availability of data for model input and the formulation and validation of processes. Data for models can originate from a wide range of sources, such as empirical lab or field studies, descriptive studies, and expert or local knowledge. Processes can be modelled based on first principles or mechanistic relationships between drivers and indicators or empirical derivatives thereof. Increasingly, the need for modelling social-ecological systems with complex feedbacks between humans and ecosystems is recognized (Downing et al. 2014; Mooij et al. 2019). Such models encompass processes and impacts of human pressures as well as interventions to mitigate the impacts on the desired ecological outcomes.

Finding an ideal model for the desired values in the NFF is difficult; a recommended approach is working towards a conceptual model (Downing et al. 2014). One way of building such a conceptual model is via the Driver-Pressure-State-Impact-Response framework (Borja et al. 2006), a framework that also explicitly includes indicators. The DPSIR framework is used in Europe for the Water Framework Directive (WFD; 2000/60/EC), and it assists managers in understanding the socio-ecological system in terms of *driving* forces (e.g. social, economic, or environmental developments), that exert a *pressure* on the ecosystem, which results in a change of the *state* of the ecosystem, which then *impacts* other elements of the socio-ecological system, and leads to *responses* of society to remedy undesirable impacts. Once a conceptual model exists, it can be used to define potential scenarios or pathways towards quantifiable end goals and aid in the selection of (more) quantitative models.

Mathematical modelling in the context of the conceptual model will require models that contain indicators for desired goals, drivers and pressures influencing said goals (directly or indirectly), and measures that can be taken to steer ecological and social developments towards reaching said goals. It is unlikely that a single mathematical model includes all elements of interest. Nor will all data needed to parameterize or validate such a model be readily available. Nonetheless, there is a broad range of freshwater ecosystem models available (Janssen et al. 2015). Repurposing existing models to fit questions posed by the developed pathways requires either: (1) adding a missing element to a pre-existing model (e.g. addition of habitat suitability curves to the HEC-RAS model in Kim and Choi 2021) or (2) connecting multiple models to form a modelling chain (e.g. connecting IMAGE and GLOBIO-Aquatic in Janse et al. 2015).

To integrate models (either directly, or via modelling chains) one can opt to either connect the models themselves or to connect the people from communities of practice around different models, and transfer inputs, outputs, and insights. Connecting models requires expertise to be concentrated in one place, but the resulting model could be optimised for scenarios and possibly be run faster without the need for continuous consultation. For connecting people across communities of practice, the expertise could remain in the places where models were developed, and due to frequent consultation, the number of errors made during the process could be reduced (Janssen et al. 2015). It is recognized that linking models can increase uncertainty (Voinov and Shugart 2013). IPBES (2016) provides a detailed discussion of uncertainty inherent in the use of scenarios and models.

When current knowledge is insufficient to formulate relationships needed to model a given pathway, either new research will be needed to elucidate these relationships (i.e. expanding the knowledge base) or the pathway would need to be abandoned as too uncertain due to lack of knowledge on key components. Both outcomes are valid and valuable, as they can focus (empirical) research or promote choices for evidence-based pathways over non-evidence-based pathways.

Knowing some starting points on freshwater ecosystem models can speed up the process of building the freshwater ecosystem models that we need to start modelling the scenarios pertaining to the NFF values perspectives. Therefore, in the next section, we will provide examples of freshwater models that are currently in use.

##### Nature for Nature models

Models for Nature for Nature typically simulate the biological integrity of water systems or their components. An example of a model that fits well in the Nature for Nature model category is GLOBIO-Aquatic (Janse et al. 2015). GLOBIO-Aquatic contains empirical relations between pressures and biodiversity and has the mean relative abundance of original species (MSA), representing biodiversity intactness, as an outcome. GLOBIO-Aquatic has been applied in a modelling chain with the integrated assessment model IMAGE, using the information on land use, hydrology, and climate, to come up with predictions on biodiversity intactness of freshwater ecosystems under different scenarios (Stehfest et al. 2014; Janse et al. 2015). An advantage of applying this model chain is that it includes the whole socio-ecological system from drivers to responses. Besides that, it is a model intended for use on a global scale (Janse et al. 2015). However, as the model is based on empirical data comparing undisturbed and disturbed ecosystems, it is less suited to be applied in semi-natural landscapes where the definition of the ‘reference state’ might be ambiguous.

Other examples that suit the Nature for Nature category well are models that simulate the habitat suitability of freshwater ecosystems. For example, the model RHABSIM was used to explore if the endangered Robust Redhorse (a fish species native to the south-eastern USA) could be reintroduced into a freshwater river reach in between two dams, by checking how the physical attributes of this river reach aligned with the spawning habitat preferences of the fish (Fisk II et al. 2014). Additionally, the combination of the process-based hydrodynamic model ELCOM and ecosystem model CAEDYM has been used to simulate how water temperature and dissolved oxygen will change under different climate scenarios and how that in turn could impact the growth or death of fish species living in cool waters (Missaghi et al. 2017). Another, more generic, tool for estimating habitat suitability is HABITAT (Haasnoot and van de Wolfshaar 2009). This tool allows the user to spatially estimate suitable habitats for a species of interest, by overlaying maps with information on habitat characteristics with knowledge rules on habitat requirements. An advantage of these habitat suitability models is that the underlying processes can be simulated via process-based models, allowing for application in all kinds of scenarios. The disadvantage of these models is that they usually focus on the ecosystem itself, and do not include societal influences directly. Besides that, the fact that a species could occur in a certain area does not necessarily mean that it will occur in this area, say in case of a hard barrier between the species and the area.

Single-species-models or assemblages of multiple-species-models are also representative of Nature for Nature, as these models aid in the understanding and protection of single or multiple species by connecting the occurrence of species to environmental factors. These models differ from the habitat suitability models in their aim to predict and understand the presence of a species, rather than the presence of its habitat (Peterson and Soberón 2012). Recent examples of freshwater multiple-species-models are that of Inoue et al. (2017), who investigated abiotic and biotic factors underlying the distribution and co-occurrence of two mussel species in European river ecosystems to improve conservation efforts, and that of Barbarossa et al. (2021), who made a global model of riverine fish species to assess possible climate-related range shifts. Examples of single species models are present in Jähnig et al. (2012), where a chain of models was used to predict the occurrence of a freshwater bivalve in a river ecosystem, and in Mafuwe et al. (2022) where the maximum entropy model MaxEnt was used to estimate the region of occurrence of three threatened Zimbabwean freshwater species for which limited data were available. As species models are more geared towards extrapolating patterns in nature, they are usually correlative (i.e. their relationships are based on empirical data), and less likely to address the direct influence or response of society upon the targeted species.

##### Nature for Society models

Models for Nature for Society typically simulate the benefits people obtain from freshwater systems and the underpinning processes, including anthropogenic impacts. For example, watershed models that simulate the runoff of water and associated constituents from the landscape can be used to represent ecosystem services in support of Nature for Society. In particular, process-based watershed models can be used to assess multiple future scenarios of climate and land use. Francesconi et al. (2016) reviewed 44 papers using the SWAT model from an ecosystem services perspective and found that the model was used to look at provisioning services such as stream flow and water yield, regulating services for water and sediment, or a combination of the two types of services. Multiple watershed models may be used together in an ensemble approach: Joint Research Centre et al. (2015) provide an example of this approach for the Danube watershed, which supports the second-largest river system in Europe. Watershed models alone do not represent the full socio-ecological spectrum, typically only linking stressors to state, with links to impact determined through post-processing of model results. However, watershed models can be used within integrated modelling approaches to support freshwater systems analysis (Li et al. 2019; Yang et al. 2022).

The provisioning of food through fisheries is another important freshwater ecosystem service, particularly from large lakes (Welcomme et al. 2010; Sterner et al. 2020). While not as substantial as for marine systems, modelling literature exists for the provisioning of food and subsistence resources in freshwater (Lorenzen et al. 2016; Natugonza et al. 2016). These models include statistical and population models, which may represent linkages to drivers and pressures, as well as management activities. There are limited examples of models for other types of food provisioning, such as plants and wildlife (e.g. Aagaard et al. 2019). Food provisioning models tend to be focused on the management of the resource, and often are not integrated with models for other ecosystem services—this is an area for future development.

Examples of integrated approaches to modelling ecosystem services, including freshwater services, and linking to human impact, are InVeST and ARIES (Vigerstol and Aukema 2011). The advantages of these two ecosystem service models are that they cover multiple provisioning and regulating services in support of Nature for Society (with Nature as Culture and Nature for Nature addressed to a lesser extent), that they consider alternative scenarios for land use and that they can be applied at different spatial scales. These and a number of other ecosystem services models include modules for economic valuation of both material and non-material benefits of nature; displaying a variety of approaches to economic valuation ranging from direct market prices to non-market valuation techniques (Dasgupta 2021). Additional ecosystem service model examples can be found in the IPBES (2016) models and scenarios assessment.

While applied models seem particularly useful for the Nature for Society perspective, we note that theoretical models too can provide key insights for understanding the dynamic dependencies of people on nature. An example is a theoretical model that outlines the trade-offs between various human uses of an ecosystem that differ in their impact on the ecosystem (Scheffer et al. 2000).

##### Nature as Culture models

Models used for Nature as Culture typically relate to arts, beliefs and other relational values of water systems. The modelling field that simulates Nature as Culture is still evolving, yet some insightful examples exist. When species are of cultural significance, models for these species are useful for estimating the impacts of scenarios on freshwater cultural services. Species distribution or species abundance can be simulated with species distribution models or population models (see “Nature for Nature models”). Similarly, other aspects of the freshwater ecosystem (not being species) that hold cultural value can be simulated with models that specialise in these aspects (e.g. freshwater ecosystem or watershed models, see “Nature for Society models”). However, for all these models, additional information on their cultural relevance will be needed to place the model outputs or inputs in the context of their cultural significance. For example, even when river discharges are accurately modelled, it will be only through interaction with indigenous and/or local people that the amount of river discharge required for religious purposes comes to light (Lokgariwar et al. 2014). As such, indigenous and local communities have an important role to play in model construction and the interpretation of model outputs.

Where some aspects of cultural values are readily modelled, other aspects, such as the values people place on a natural area, are more difficult to capture in numbers. In some cases, these values can become part of the modelling process, e.g. through model selection or the prioritisation of model outcomes by stakeholders via a participatory modelling approach (Voinov and Gaddis 2008). In other cases, the cultural values appear intangible; for how do we model values such as harmony with nature, inspiration, or the loss of local ecological knowledge? A proxy for some of these values could be the intactness and functioning of nature itself, and thereby in the models that fall into the Nature for Nature category (see “Nature for Nature models”). For there appears to be a positive relationship between aesthetic appreciation and the intrinsic values of nature (Arias-Arévalo et al. 2017). Another proxy could be the access people have to an area. Models that simulate access to areas are used in the tourism industry, by environmental economists, and by spatial planning agencies. For example, a model created to estimate the use of wildlife parks in Africa based on entrance fees (Day 2000), might be adapted to estimate the accessibility of nature to different groups of people. Additionally, models for non-economic valuations of nature (e.g. Maher et al. 2020), even though they are inherently grounded in the Nature for Society perspective, might provide inspiration for modelling the Nature as Culture perspective.

## Discussion

### Towards pluralistic freshwater nature futures

**Fig. 2:**
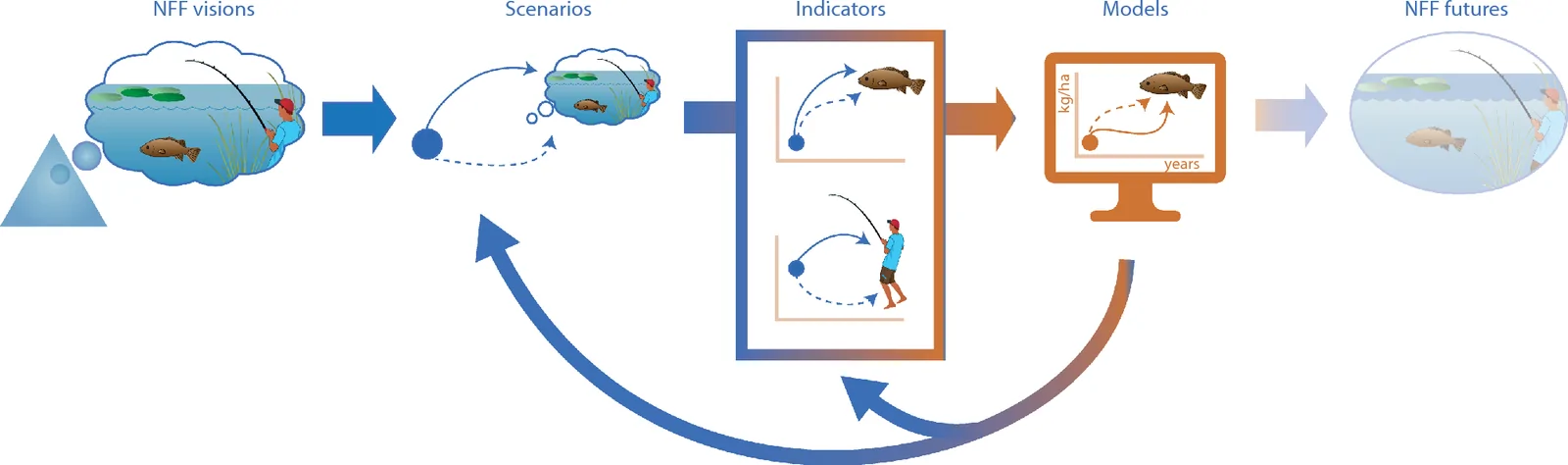
With the NFF we are now able to create positive and holistic visions of the freshwater ecosystem futures we want (NFF visions). Operationalizing these futures requires us to develop scenarios that lead us from the present to our visions (Scenarios; the dark blue dot represents the current state of our freshwater ecosystems, and the arrows represent the different pathways we could take to reach our desired future). The scenarios we design are frequently abstract. To make them tangible, we have to define which elements of our future are measurable or quantifiable, i.e. which elements are our indicators (Indicators). Once we know our indicators, we have a bridge between our scenarios and models, and we can start approximating the impacts of our ideas with models (Models; the bridge between qualitative and quantitative is indicated with the colour gradient from blue to orange). Working from scenarios to models is not a linear process, but rather an iterative process (as indicated by the arrows going back from the models to the scenarios picture). With the model outcomes we can start to formulate actions and bring our ideas to life, thus bringing us to the NFF futures we want (NFF futures; this picture is transparent, as the future always remains uncertain). The environmental vector images were sourced and adapted from Integration and Application Network (ian.umces.edu/media-library)

Freshwater modelling can be linked to the NFF by generating scenarios to drive the models (Fig. 2). The three value perspectives in the corners of the triangular NFF provide a minimal set of entry- and anchor points for developing diverse social-ecological scenarios (Pereira et al. 2020). The resulting nature futures do not necessarily need to reflect these three value perspectives, as in fact, the corners provide extreme perspectives on what can still be considered ‘desirable’ (Durán et al. 2023). In practice, we expect many nature futures scenarios to be a mix of the value perspectives. Yet, focusing on the three perspectives helps to explain how clearly contrasting nature futures scenarios can be produced from the NFF. Especially when quantified, the analysis of these scenarios will expose the consequences of alternative development trajectories, including inevitable trade-offs, but also possible synergies. Therefore, and because the three perspectives are a distinctive part of the NFF, we organised the structure of this paper around the three archetypes. At the same time, the presentation of relevant concepts in “Unpacking the value perspectives on freshwater nature futures”, and indicators of Box 2, showed how the three value perspectives can be linked to each other through the concepts, and hence the policy options related to these concepts. The three perspectives can subsequently be used as entry points for further consideration of the interior of the triangular space for the development of nature futures scenarios that enhance multiple values of nature simultaneously. While tensions may be expected within a nature futures scenario where any of the value perspectives blend, identifying these tensions is a key step towards mitigating them to achieve the desired future. For example, knowing that dam removal for nature restoration in long-humanized landscapes can jeopardize people’s identity and relationship with the landscape (Fox et al. 2016), aids in identifying either active stakeholder engagement or alternatives to the dams’ removal as new steps forward (Habel et al. 2020). Importantly, the methodological approach that we outlined for developing freshwater nature futures remains generally the same.

### Building the bridge to freshwater modelling communities

From the examples of the freshwater ecosystem models available to each value perspective, it is clear that the current state-of-the-art of aquatic ecosystem modelling has developed a plethora of useful outcomes for aquatic ecosystem responses to varying pressures, providing support for Nature for Nature. These outcomes have also been expressed in terms of their impact on ecosystem service provisioning, or are framed as such, which provides partial support for Nature for Society. However, there are limited examples of freshwater ecosystem models supporting Nature as Culture. The challenge is thus to bridge these gaps and to start incorporating the full range of value perspectives of the NFF into our freshwater ecosystem models. A way forward here is to start introducing the NFF to the forums of the freshwater modelling communities (e.g. AEMON at https://groups.google.com/g/aquaticmodelling?pli=1, GLEON at https://gleon.org/, ISIMIP at https://www.isimip.org/, UN EP WWQA at https://communities.unep.org/display/WWQA). Bringing the NFF into these communities will also start the process of shifting our focus from only modelling the undesirable futures we ‘might end up with’, towards the futures we want to achieve.

Building a bridge between the NFF and the freshwater modelling communities will not only be beneficial to freshwater modelling efforts but will also aid the development of the NFF and its nature futures. Modellers, by the definition of their work, are forced to be logically consistent. Hence, in a setting where the NFF is applied, modellers are likely to make sure that futures are defined with plausible and/or quantitative indicators. Also, gaps in knowledge, tools and expertise may be defined earlier when tackling ‘defining pathways towards desirable futures’ together. Involving modellers early on in the design and implementation of the NFF to case studies will ensure that model outcomes, value perspectives and quantification thereof become integrated (Volkery et al. 2008).

### Aligning models and data for freshwater futures

Developing tools that answer relevant questions, inform policy and further scientific research requires a co-creation effort between empirical scientists, stakeholders and modellers. Sharing data via open repositories in a FAIR manner is essential to stimulate model development and validation (Wilkinson et al. 2016). Although data parameterisation and the process formulation of models are firmly in the realm of the modelling community to derive, identifying their need is a joint effort. Further, gathering the data to adequately parameterize such processes will require interdisciplinary experts to ensure suitable designs of experiments and measurements. While data for both validation and parameterization may be lacking to model all aspects of the NFF at current, the application of the NFF can help to identify and focus research efforts. Co-creating NFF pathways with modellers will help to anchor processes relevant to desired pathways firmly into ongoing model development, increasing model applicability to environmental policy questions.

### Ways forward

Perhaps the largest challenge is representing the complexity of freshwater ecosystems in the context of the socio-ecological system. To advance the field of modelling of pathways towards desirable futures we will need to push the boundaries of transdisciplinary modelling efforts towards true social-ecological models. Social-ecological models are currently in their infancy (Downing et al. 2014; Hughes et al. 2017; Sun and Hilker 2020) and including feedback between societal actions and perception on top of ecological responses in future models is acknowledged as an important step forward (Mooij et al. 2019). Doing so will require integrated models, where not only societal drivers and pressures are modelled with respect to their ecological outcomes, but where resulting ecological outcomes will change societal actions accordingly. Designing, constructing and testing such models will need to reach across disciplines, a time-consuming and non-trivial process, but one that has borne fruit before, e.g. through integrating physical and biological components (Saito et al. 2009; Guswa et al. 2020). We envision two concrete steps towards such integrated models: (1) connecting existing models to explore the current reach of existing tools and (2) applying and integrating the NFF in the development and set-up of future freshwater modelling efforts.

#### Connecting existing models

Connecting already existing models by finding common denominators between them on which they can connect, can help push the field forward towards more integrative modelling. For instance, models of nutrient runoff may be connected to ecological models using nutrient loads as inputs (Li et al. 2019). Similarly, ecosystem service provisioning models can be connected to ecological models through the ecological outcomes the services depend on. Doing so allows for linking existing models to start with predicting varying aspects of the value perspectives. Working with different connected models will allow the field to gain insights into aspects of propagation impacts as well as their respective uncertainties among model components (Tscheikner-Gratl et al. 2019). Optimising such separated chains of models towards a predefined target will prove challenging when the models are not integrally connected (i.e. run separately and sequentially). To tackle this challenge, defining intermediate targets corresponding to each model can offer a solution to still perform an optimization, though this is more cumbersome than in a fully integrated model. Nonetheless, connecting existing models makes the most of already existing tools and knowledge, and could serve as a platform to work toward interdisciplinary teams by connecting model experts. Existing calls to unite efforts for freshwater biodiversity science and conservation [i.e. ‘Alliance for Freshwater Life’ (Darwall et al. 2018), the ‘Emergency Recovery Plan’ (Tickner et al. 2020), the ‘Recommendations for Safeguarding Freshwater life beyond 2020’ (van Rees et al. 2021)] and the IPBES invitation to modelling communities around the world should be encouraged to try out the Nature Futures Framework (Decision IPBES-9/1), and will offer platforms to combine existing freshwater models and explore their application domain in the context of the NFF.

#### Applying the NFF to freshwater policy and management

The NFF, and the models designed within its wake, can be applied to freshwater policy and management at global to local spatial scales to support desired nature futures. This would be in line with Feio et al. (2021), who identified the need for establishing common collaborative frameworks for managing international river catchments.

Applying the NFF to freshwater management and policy at the global scale can ensure that a plurality of value perspectives is considered while leaving room for local modelling efforts to specify such perspectives in more detail. We foresee that the NFF will inform global studies that focus on freshwater nature and biodiversity in general, on global drivers of change, on supporting and regulating ecological services (e.g. food provision, freshwater provision, nutrient retention, greenhouse gas emissions) and preserving transnational cultural heritage, such as rivers. Global studies may find that the inclusion of certain value perspectives is challenging since the valuation of ecosystem services and biodiversity vary both regionally and culturally. For even though cultural relationships with nature exist all around the globe, they have no single denominator. Participation of diverse governmental and non-governmental stakeholders and community representatives will be needed to expand freshwater policy at global or national scales to ensure the adoption and integration of NFF perspectives into coherent legislative frameworks.

At the local or regional scale, the NFF can be instrumental in refining watershed and water body management. Water and nature managers as well as local inhabitants will be necessary to co-design eco-centric waterscapes and management strategies that ensure sustainable future water quality for nature and people in a region. We foresee that local studies will be able to focus on specific nature conservation targets, provisioning ecosystem services and the expression of the regional and local cultural values of nature. The NFF could aid in developing collaborative river restoration goals and targets, which are critical for success (Angelopoulos et al. 2017). The application of NFF can also be used to identify trade-offs among management options (Palacios-Abrantes et al. 2022). Modelling of freshwater systems in the NFF context could support the characterization of the full range of projected impacts for permitting development activities in aquatic systems. Chen and Olden (2017) applied a similar approach to the management of a dam-regulated river.

Connecting local and global freshwater modelling approaches within the context of the NFF ensures interoperability, in that they mutually make use of each other’s insights, both scaling up and scaling down, and supports a shared consciousness of the plurality of nature across spatial scales. But, ultimately, the proof of the pudding is in the eating. Practical modelling applications of the NFF are needed to learn from experience and build on each other’s work. We hope that our article will provide an important impetus in this regard.

## Conclusion

In our exploration of the NFF for freshwater ecosystem models we have found that the NFF aligns with several current concepts in freshwater ecology. Furthermore, we can translate the NFF to freshwater systems through scenarios, with freshwater futures characterised by indicators. We also found that existing freshwater ecosystem models can benefit from the use of the NFF by characterising freshwater processes and pathways, and quantifying the effects of alternative scenarios. Current freshwater ecosystem models can represent Nature for Nature, partially represent Nature for Society, and represent limited or indirect aspects of Nature as Culture. We believe that the knowledge gaps that exist should be bridged by mobilising existing freshwater research communities and projects to model nature futures for freshwater ecosystems. We see the development of the Nature Futures Framework under IPBES as an opportunity for freshwater modelling communities from across the world to strengthen the representation of freshwater nature and biodiversity in global environmental governance. At the same time, we believe that innovations and novel collaborations are necessary to fully operationalize this new scenario framework for it to deliver on the promise of guiding human societies towards desirable futures for people and nature. We, therefore, conclude with a call to action*: “Only by joining forces and expertise can we solve the global freshwater biodiversity crisis*.”
